# The Role of Narcissism and Dissociation in the Indirect Relationship Between Childhood Trauma and Body Dissatisfaction, Bulimia, and Drive for Thinness in a Sample of Adolescents: A Path Analysis Model

**DOI:** 10.1007/s40653-026-00958-4

**Published:** 2026-08-08

**Authors:** Fabio Carraturo, Marzieh Abdoli, Dua Fatima Afzaal, Paolo Cotrufo, Stefania Cella

**Affiliations:** Department of Psychology, University of Campania “Luigi Vanvitelli”, Caserta, Italy

**Keywords:** Childhood trauma, Body dissatisfaction, Bulimia, Drive for thinness, Dissociation, Narcissism, Adolescents, Path analysis

## Abstract

This study examined the associations between childhood trauma, body dissatisfaction, bulimia, and drive for thinness in adolescents, with a specific focus on the roles of narcissistic traits and dissociative experiences as intervening variables. A sample of 439 high school students from Southern Italy completed self-report measures assessing eating-disorder-related symptoms, pathological narcissism, childhood trauma, and dissociation. The hypothesised path model showed significant indirect associations between emotional abuse and both drive for thinness and bulimia through vulnerable narcissism and dissociative experiences. More specifically, depersonalisation was positively associated with bulimia, whereas passive influence was positively associated with drive for thinness. Grandiose narcissism showed a negative association with body dissatisfaction and was not positively associated with the eating-related outcomes. Multi-group analyses indicated that the model was not invariant across males and females. In males, absorption was involved in the indirect association between childhood trauma and body dissatisfaction, whereas this pathway was not observed in females. These findings suggest that childhood trauma may be linked to eating-disorder-related features through different psychological pathways involving narcissistic vulnerability, specific dissociative dimensions, and gender. Because of the cross-sectional design, the findings should be interpreted as associative rather than causal.

**Supplementary Information:**

The online version contains supplementary material available at https://doi.org/10.1007/s40653-026-00958-4.

## Introduction

The aetiology and presentation of eating and body related symptoms are complex and involve a plurality of biological, environmental, and psychological factors (Hilbert et al., 2014; Solmi et al., 2016). Among these, childhood trauma has been consistently identified as a nonspecific risk factor for the development of clinical and subclinical eating syndromes, but particularly of the binge-purge type (Barakat et al., 2023; Dube et al., 2001; Mahon et al., 2001; Steinhausen, 2020). Childhood trauma, defined as “unbearable and inescapable single or continuative threatening experiences in the face of which a person is powerless” (Farina et al., 2019, p. 4), involves early occurrences of physical, emotional, and sexual abuse and neglect, which have a substantial influence on a child’s physical and mental health, with long-term psychological consequences (Briere et al., 2008; Muskett, 2014; Tzouvara et al., 2023). These heterogeneous manifestations of trauma are considered critical diatheses for severe forms of psychological distress, owing to their substantial and lasting impact on attachment (Farina et al., 2019) emotional regulation (Al Odhayani et al., 2013; Briggs et al., 2021; Felitti et al., 1998), identity development (Hayes et al., 2021; Lewis et al., 2021), and personality structure (Van der Hart & Dohary, 2023; Sar, 2017). These vulnerabilities can contribute to an increased development of psychological characteristics associated with eating disorders. Childhood trauma has been consistently linked to body dissatisfaction (Bödicker et al., 2022; Momeñe et al., 2023). Additionally, some researchers have found evidence for more specific relationships between the type of trauma and eating or body related outcomes. Physical neglect has been previously associated with body image and bulimic symptoms (Kong & Bernstein, 2009). Sexual abuse survivors, who often have distorted beliefs of responsibility for their abuse, may come to feel ashamed of their body and attempt to render it unattractive or punish it. This can manifest as intense body dissatisfaction and as an excessive desire for thinness (Rabito-Alcón et al., 2021). Emotional abuse has been instead particularly linked to bulimia (Gerke et al., 2006; Groleau et al., 2012). Other studies have reported less distinct effects of all kinds of trauma with desire for thinness, bulimic tendencies, and body dissatisfaction (Monteleone et al., 2020).

Attention has been called to the investigation of moderators intervening in the effect of childhood abuse and neglect on body image disturbances, and dissociation has been proposed as a possible intermediate link (La Mela et al. 2010; Rabito-Alcón et al. 2020a; Cipriano et al. 2024). According to the most widely supported explanatory models of dissociation, dissociative states can be understood as sequelae of past traumatic events (Dell, 2023; Farina et al., 2019; Lynn et al., 2019; Maldonado & Spiegel, 2008). Dissociative tendencies also appear highly prevalent in people with eating and body related symptoms (Longo et al., 2023; Lyssenko et al., 2018), and in combination with such symptoms they increase the occurrence of self-harm, suicidal attempts, negative therapeutic outcomes, and a history of traumatic events (La Mela et al., 2010; Longo et al., 2023). The disjunction between body and mind that can derive from a dissociative breakdown of normal integrative processes (O. Van der Hart & Dorahy, 2023) is relevant to key features involved in the onset and maintenance of eating pathology, prompting people to focus on the regulation of their bodies and physiological needs as a means to cope with underlying psychological distress (Engelberg et al. 2007; Kluft 2001; Palmisano et al. 2018; Rabito-Alcón et al. 2020a). Dissociation has in fact been linked to desire for thinness, bulimia, and body dissatisfaction in adolescents (Quattropani et al., 2022). However, since recent evidence points towards dissociation being a multidimensional construct (Briere et al., 2005; Ellickson-Larew et al., 2020), these findings require a more minute understanding of the specific subdimensions involved in dysfunctional attitudes towards the body.

Pathological narcissism has also been claimed to have a trauma-related aetiology (Rosen et al., 2021; Şar & Türk-Kurtça, 2021), in the form of caregivers’ harsh criticism (Masterson, 1993), unrealistic valuation (Nguyen & Shaw, 2020) and inadequate mirroring (Fonagy et al., 2002). In fact, both narcissism and dissociation have been posited to reflect a common deficiency of the self-system, owing to the disorganising failures of the child’s early environment (Kluft, 2018). Premature invalidation of the child’s mental states is thought to inflict of a “narcissistic wound”, i.e. a primitive deficiency of the child’s basic trust towards his caregivers and consequently towards his own self-worth and security, which in turn can result in a state of self-disintegration with dissociative features (Ronningstam & Maltsberger, 1998; Şar & Türk-Kurtça, 2021). Notably, narcissism can be further categorised into two distinct subtypes: grandiose and vulnerable (Miller et al., 2017). Overt displays of superiority, self-assurance, and entitlement characterise grandiose narcissism. Individuals exhibiting grandiose narcissism often safeguard their self-esteem by dismissing unfavourable input from others and hold a defensively exaggerated sense of self-worth (Krizan & Herlache, 2018; Pincus et al., 2009). Conversely, vulnerable narcissism encompasses an acute need for validation, heightened sensitivity to external judgments, diminished and contingent self-esteem, and pervasive insecurity with a consequent proneness to hide devalued aspects of oneself (Brewerton, 2007; Hyatt et al., 2018; Klein et al., 2022; Masterson, 1993; Şar & Türk-Kurtça, 2021). Moreover, narcissistic traits have been repeatedly observed to play a role in dysfunctional body related attitudes (Barakat et al., 2023; Hilbert et al., 2014). People exhibiting narcissistic traits depend on external validation, such as praise or success, to boast their self-worth and may manipulate their bodies and dietary practices to attain an ideal of perfection, hence increasing the likelihood of eating disorder symptoms (Boone et al., 2012; Costanzo et al., 2022; Gaudio et al., 2016; Lloyd et al., 2015). Consistently with this view, narcissism has shown associations with desire for thinness (Szymczak et al., 2023), and bulimic tendencies (Littrell et al., 2021). A link between narcissism and body dissatisfaction has also been evidenced (Hater et al., 2021), but interestingly the two narcissistic subtypes display opposite effects in relation to this construct. Whereas grandiosity seems to reduce body dissatisfaction, narcissistic vulnerability heightens it (Carrotte & Anderson, 2019; Purton et al., 2018).

The fragility which is apparently compensated in grandiose narcissism but is instead evident in the vulnerable narcissistic subtype can be understood as an inability to integrate self-experiences into an authentic whole (Şar & Türk-Kurtça, 2021; Schimmenti & Sar, 2019). In more general terms, a fragility of the personality structure of the subject has been proposed as a potential factor contributing to the relationship between adverse life events and dissociation through complex and non-linear effects (Maldonato et al., 2018). Failure to synthesise in a coherent totality one’s own psychological activity may result from a disrupted early developmental history and may constitute a fundamental process distinguishable from but preparatory of dissociative phenomena (Farina & Meares, 2022). Dissociation can be thus described as a compartmentalised plurality of mental states, contents and functions arising from trait disintegrative vulnerability (Farina & Meares, 2022; Moskowitz & van der Hart, 2020). This is consistent with Hughling-Jackson and Janet’s models of the mind and of mental disorders. In line with the historical contributions of these authors, psychopathology can be conceptualised as invariantly expressing an alteration of the integrative function of consciousness, a function which, when deficient, can no longer modulate the emergence of impulsive or automatic intrusions into thought and action (Farina et al., 2005; Meares, 1999; Van der Kolk et al., 1989).

Taken together, this theoretical framework and the empirical evidence reviewed suggest that posttraumatic dissociative tendencies driven by a narcissistic organisation can lead adolescents to displace unfulfilled emotional demands onto external regulating elements, such as their physical appearance, or onto their bodily functions in an attempt to offset uncontrollable impulses and negative affects (Clemens et al., 2022; Day et al., 2024; Young & Widom, 2014).

Prior research has investigated these factors separately; however, the roles of narcissistic traits and dissociative experiences in the indirect association between childhood trauma and body dissatisfaction, bulimia, and drive for thinness have not been comprehensively examined within a single model (Day et al., 2022; Yilmaz et al., 2015). The present model was specified a priori on theoretical and developmental grounds. Childhood trauma was conceptualised as an early adverse developmental exposure, narcissistic traits as dimensions of self-organisation and personality vulnerability, dissociative experiences as disruptions in psychological integration and self-regulation, and body dissatisfaction, drive for thinness, and bulimia as current eating-disorder-related outcomes. This ordering was theoretically driven and should not be interpreted as evidence of causality. The present study therefore tested whether childhood trauma was indirectly associated with body dissatisfaction, drive for thinness, and bulimia through grandiose narcissism, vulnerable narcissism, and dissociative dimensions. We expected childhood trauma, particularly emotional abuse, to be positively associated with vulnerable narcissism and dissociation. We also expected vulnerable narcissism to show stronger positive associations with dissociative experiences and eating-disorder-related outcomes than grandiose narcissism. Finally, because dissociation is a multidimensional construct, we expected the A-DES dimensions of amnesia, depersonalisation, passive influence, and absorption to show different associations with body dissatisfaction, drive for thinness, and bulimia. Gender differences were examined through multi-group analysis.

## Materials and Methods

### Procedure

All variables were measured with a battery of self-report questionnaires. Participants were 452 students recruited in high schools located in southern Italy. The headmaster’s approval was granted prior to administration and informed consent from the student’s guardians was obtained. The research protocol was administered during regular school hours under the supervision of a teacher and a research assistant.

### Measures

#### Demographic Sheet

Participants completed sociodemographic sheets informing regarding their age, sex, self-reported weight and height, nationality, number of family members, relationship status, sexual orientation, education and relationship status of the parents, frequency of physical exercise, presence of tattoos, piercings or cosmetic surgery.

#### Eating Disorder Inventory-3

Eating Disorder Inventory-3 (Garner, 2004) provides comprehensive measures for the psychological and behavioral traits of eating disorders, including body dissatisfaction, bulimia, and perfectionism. In keeping with the objectives of the present study, only the three eating disorder-specific sub-scales measuring eating disorder risk were administered. These sub-scales measure the drive for thinness, bulimia and body dissatisfaction. The EDI-3 has been used with adolescent samples and is appropriate for participants aged 13 years and older according to the instrument manual (Garner, 2004). We used only the three eating-disorder-risk subscales because they directly corresponded to the outcomes of interest. In the present sample, internal consistency was good for Drive for Thinness (Cronbach’s α = 0.88, McDonald’s ω = 0.91), Bulimia (α = 0.82, ω = 0.88), and Body Dissatisfaction (α = 0.84, ω = 0.88).

#### Brief Pathological Narcissism Inventory

The Brief Pathological Narcissism Inventory (B-PNI) is a self-report questionnaire developed to measure pathological narcissism (Wright et al., 2010). Its short form (Schoenleber et al., 2015) comprises 28 items rated on a 6-point Likert scale designed to measure grandiose narcissism and vulnerable narcissism on seven facets: Exploitativeness, Fantasy, Self-sacrificing, Devaluing, Self-enhancement, Hiding the self, Entitlement rage. The use of the B-PNI in adolescents is supported by psychometric evidence from Italian high-school students, showing adequate properties for the assessment of pathological narcissism dimensions in adolescent samples (Somma et al., 2020). In this sample, internal consistency was good for Grandiose Narcissism (α = 0.79, ω = 0.84) and Vulnerable Narcissism (α = 0.90, ω = 0.91).

#### Childhood Trauma Questionnaire-short Form

Childhood trauma Questionnaire Short Form (CTQ-SF; Bernstein et al., 2003) is a 28-item scale designed to assess five types of childhood trauma: emotional abuse, physical abuse, sexual abuse, emotional neglect and physical neglect. It assesses the items on a 5-point Likert type frequency scale with scores ranging from 1 (never) to 5 (always). This scale provides a comprehensive understanding of childhood maltreatment on both dimensional and categorical levels. The CTQ-SF has been used in adolescent clinical and community samples, with evidence supporting its use for assessing childhood maltreatment during adolescence (Hagborg et al., 2022). Because the present sample included participants aged 13 to 18 years, the age range of the instrument was considered appropriate, although the use of retrospective trauma questionnaires in younger adolescents remains a methodological point that should be considered when interpreting the findings. For this sample, internal consistency was acceptable to excellent for Physical Abuse (α = 0.84, ω = 0.88), Emotional Abuse (α = 0.85, ω = 0.87), Emotional Neglect (α = 0.84, ω = 0.87), and Sexual Abuse (α = 0.92, ω = 0.94). Cronbach’s alpha was lower for Physical Neglect (α = 0.60), although its McDonald’s omega was acceptable (ω = 0.71).

#### Adolescent Dissociative Experiences Scale (A-DES)

The A-DES (Armstrong et al., 1997) is a 30-item self-report scale designed to measure the frequency of dissociative experiences in adolescents. The questionnaire uses a 10 point Likert frequency scale, with scores ranging from 0 (never) to 10 (always). The A-DES addresses four dimensions of dissociation: amnesia, passive influence, depersonalisation and absorption. Amnesia is defined as dissociative disturbances of autobiographical memory for self-relevant events and information. Passive influence is instead characterised by the feeling that one’s behaviours are caused by an ego-alien force from within. Absorption can be described a state of intense focus and involvement in internally generated states. Finally, depersonalization/derealisation indicated a loss of the sense of familiarity or ownership of one’s body, as well as by a sense of alienation from one’s mental or physical world. The A-DES was specifically developed for adolescents, and the Italian version has shown a four-factor structure consistent with the original subscales in adolescent and young adult samples (Armstrong et al., 1997; De Pasquale et al., 2016). For this work, internal consistency was acceptable to good for Amnesia (α = 0.80, ω = 0.84), Depersonalisation/Derealisation (α = 0.70, ω = 0.75), and Passive Influence (α = 0.78, ω = 0.81). Cronbach’s alpha was slightly below 0.70 for Absorption (α = 0.68), although McDonald’s omega was acceptable (ω = 0.75).

#### Data Analysis

Analysis of missing data revealed that 7.2% of our variables had missing values, which amounted to 2.2% of the total dataset and were missing completely at random (Little’s MCAR: χ²=28643.9; df = 28700; *p* =.591), therefore a multiple imputation with five iterations was performed using a predictive mean matching method (PMM). All variables later included in our model were also included in the analysis and imputation of missing data (Austin et al., 2021). Descriptive statistics and zero-order correlations were obtained for each variable and the relationship between childhood trauma, vulnerable and grandiose narcissism, sub-dimensions of dissociation and eating disorder risk was tested via a path analysis, which is an effective statistical approach to test the chained influences of several predictors and indirect effects in models which are too complicated for more straightforward analyses such as multiple regression (Streiner, 2005). Model fit was evaluated based on Chi-square statistic (χ²), root-mean-square error of approximation (RMSEA), standardised root-mean-square residual (SRMR), comparative fit index (CFI), and Tucker–Lewis index (TLI). A non-significant χ², CLI and TLI values greater than 0.90, and SRMS and RMSEA values lower than 0.08 are considered indicative of an acceptable model (Barrett, 2007; Hu & Bentler, 1999). The Maximum Likelihood estimation method was used, and total direct and indirect effects were estimated with a bootstrap procedure, where 95% confidence intervals for 5000 resampling estimates were obtained (Preacher & Hayes, 2008). Indirect effects can be considered statistically significant when the lower and upper bound of their corresponding confidence interval does not include zero (Hayes, 2017). Based on theoretical considerations and on previous findings of absent direct effects of childhood maltreatment on eating symptomatology when dissociation was inserted as an intermediate variable (Rabito-Alcòn et al., 2020b; Moulton et al., 2015; Kent et al., 1999), no direct effect of the CTQ subscales on the outcome variables was modelled. A second model including the direct effect of childhood maltreatment on eating symptomatology was then fitted and the two models were compared with an ANOVA and by evaluating the respective AIC and BIC indexes, whose lower values indicate a better fit to the data (Kuha, 2004). Sex and age were controlled as covariates except for the EDI subscales, where sex was inserted as a predictor given that eating disorder related traits are well-documented to be more prevalent among individuals of the female sex (Breton et al., 2023), and for vulnerable and grandiose narcissism, where both sex and age were inserted as predictors. Research has indeed shown that narcissism is typically expressed differently across sexes and tend to vary substantially across the lifespan, with noticeable differences even across teenage years (Orth et al., 2024; Weidmann et al., 2023).

The ordering of variables in the path analysis model was built on prior empirical evidence and theoretical assumptions. Childhood trauma and the quality of parental relationships have been shown to affect narcissistic traits and dissociation in longitudinal studies (Brummelman et al., 2015; Cohen et al., 2005; Schalinski et al., 2016). Narcissistic traits have also emerged as prospective predictors of eating disorder symptoms (Dakanalis et al., 2016; Eielsen et al., 2022). Theoretically, a narcissistic personality organization can be considered as an early developmental outcome and as such was believed to precede the emergence of later mental symptoms, while dissociation was considered conducive to eating disorder-related traits as it hinders a coherent sense of self and agency, which are often recognized as core psychological vulnerabilities in eating disorders (Şar & Türk-Kurtça, 2021; Lynn et al., 2022; Longo et al., 2023). Longitudinal studies partly confirm these assumptions, showing that dissociation can lead to eating disorder-related behaviours in momentary associations (Crosby et al., 2017). Nonetheless, longitudinal evidence is scant, and most reported relationships are complex, with longitudinal studies often reporting bidirectional temporal effects (Crosby et al., 2017; Eielsen et al., 2022). While this constitutes an important limitation, parsimony and a theoretically guided construction of the model were ultimately given priority.

Although this practice is not universally agreed upon and is only marginally informative, an alternative model with an inverse causal chain was also fitted, as this can provide some tentative indication to identify the more suitable among multiple theoretically plausible model specifications (Kim et al., 2019).

Finally, the gender invariance of the model was tested by constraining parameters across males and females and comparing the resulting model with an unconstrained one, thus obtaining a Chi-Square Difference statistic (Δχ²) for the difference of fit between the two models. Missing values analysis, multiple imputation, and descriptive analysis were performed on IBM SPSS (v.29.0.1.0), while path analysis and multi-group analysis were conducted on R using the lavaan package (Rosseel, 2012).

## Results

### Descriptive Analyses

Of the 452 participants initially recruited, 13 were excluded because of incomplete protocols (participants abandoned the administration or left at least one questionnaire entirely blank).

The final sample comprised 439 participants. Sex was available for 438 participants, of whom 217 were females and 221 were males. The age of the sample ranged from 13 to 18 years, with a mean of 15.9 (SD = 1.71). Almost the totality of our sample was of Italian nationality (95.2%). The majority of participants (90.2%) had siblings, and 89.5% were heterosexual, 2.5% were homosexual, 5.2% bisexual and 1.4% pansexual. A significant difference in age was observed between males (M = 15.31; SD = 1.51) and females (16.47; SD = 1.71), t_(436)_=−7.4, *p* <.001. Significant differences were also found between males’ and females’ means of emotional abuse (M_m_=7.9, SD_m_=3.8; M_f_=9.0, SD_f_=4.5; t_(436)_=−2.9, *p* =.004), vulnerable narcissism (M_m_=1.9, SD_m_=1.0; M_f_=2.4, SD_f_=1.0; t_(436)_=−5.2, *p* <.001), passive influence (M_m_=3.1, SD_m_=2.1; M_f_=3.7, SD_f_=2.4; t_(436)_=−2.9, *p* =.004), drive for thinness (M_m_=6.7, SD_m_=6.4; M_f_=12.1, SD_f_=8.9; t_(436)_=−7.3, *p* <.001), bulimia (M_m_=3.7, SD_m_=4.9; M_f_=6.4, SD_f_=6.8; t_(436)_=−4.8, *p* <.001), and body dissatisfaction (M_m_=9.6, SD_m_=8.4; M_f_=16.5, SD_f_=9.3; t_(436)_=−8.1, *p* <.001). Cutoffs for traumatic experiences were applied according to Bernstein and Fink (1998), who consider to be representative of moderate levels of trauma a score ≥ 15 for emotional abuse, ≥ 12 for physical abuse, ≥ 12 for sexual abuse, ≥ 17 for emotional neglect, and ≥ 12 for physical neglect. Of the total sample, 17.8% (33 males, 45 females) reported a moderate degree or higher of emotional abuse, 16.4% (36 males, 36 females) of emotional neglect, 13.7% (30 males, 30 females) of physical abuse, 16.9% (41 males, 33 females) of physical neglect, and 12.3% (33 males, 21 females) of sexual abuse. Means and standard deviations of all variables are reported in Table 1, while correlations are shown in Table 2.

**Table 1:** Means and Standard Deviations for Variables Included in the Model and Mean Differences Between Males and Females

	Total *N* = 439	Males *N* = 221	Females *N* = 217
	M(SD)	M(SD)	M(SD)
Age	15.9 (1.7)	15.3 (1.5)	16.5 (1.7)***
Physical abuse	6.49 (3.1)	6.5 (2.9)	6.5 (3.3)
Physical neglect	7.3 (2.7)	7.6 (2.9)*	7.1 (2.6)
Emotional abuse	8.4 (4.2)	7.9 (3.8)	9.0 (4.5)**
Emotional neglect	9.7 (4.5)	9.6 (4.3)	9.8 (4.7)
Sexual abuse	6.1 (3.2)	6.2 (3.1)	6.0 (3.3)
Narcissistic grandiosity	2.8 (0.8)	2.8 (0.9)	2.8 (0.8)
Narcissistic vulnerability	2.1 (1.0)	1.9 (1.0)	2.4 (1.0)***
Amnesia	3.0 (2.1)	3.0 (2.0)	3.1 (2.2)
Depersonalisation	3.1 (2.0)	2.9 (1.9)	3.3 (2.1)
Passive influence	3.4 (2.3)	3.1 (2.1)	3.7 (2.4)**
Absorption	3.2 (1.9)	3.1 (1.8)	3.3 (2.1)
Drive for thinness	9.4 (8.2)	6.7 (6.4)	12.1 (8.9)***
Bulimia	5.0 (6.1)	3.7 (4.9)	6.4 (6.8)***
Body dissatisfaction	13.0 (9.5)	9.6 (8.4)	16.4 (9.3)***

Sex was available for 438 participants; therefore, gender comparisons are based on 221 males and 217 females. Difference significant at: *p <.05, **p <.01, ***p <.001

**Table 2:** Zero-Order Correlations Between Variables Used in the Path Analysis Model

Variable	1.	2.	3.	4.	5.	6.	7.	8.	9.	10.	11.	12.	13.	14.
1.CTQ_PA	-	0.57***	0.59***	0.46***	0.65***	0.08	0.21***	0.26***	0.28***	0.25***	0.20**	−0.01	0.12	0.06
2.CTQ_PN	0.57***	-	0.43***	0.61***	0.46***	−0.04	0.07	0.25***	0.23***	0.18**	0.16*	−0.02	0.09	0.00
3.CTQ_EA	0.59***	0.43***	-	0.55***	0.41***	0.19***	0.34***	0.37***	0.45***	0.42***	0.32***	0.12	0.26***	0.21***
4.CTQ_EN	0.46***	0.61***	0.55***	-	0.32***	−0.03	0.14**	0.17**	0.24***	0.20***	0.15*	0.02	0.15*	0.11
5.CTQ_SA	0.65***	0.46***	0.41***	0.32***	-	0.08	0.16***	0.20***	0.24***	0.20**	0.13	0.01	0.06	0.01
6.PNI_GRD	0.08	−0.04	0.19**	−0.03	0.08	-	0.57***	0.22***	0.28***	0.26***	0.24***	0.15*	0.11	0.11
7.PNI_VLN	0.21***	0.07	0.34***	0.14	0.16*	0.57***	-	0.41***	0.48***	0.48***	0.37***	0.36***	0.32***	0.40***
8.ADES_AM	0.26***	0.25***	0.37***	0.17***	0.20***	0.22***	0.41***	-	0.77***	0.74***	0.77***	0.16***	0.23***	0.25***
9.ADES_DEP	0.28***	0.23***	0.45***	0.24***	0.24***	0.28***	0.48***	0.77***	-	0.83***	0.74***	0.24***	0.30***	0.28***
10.ADES_PI	0.25***	0.18***	0.42***	0.20***	0.20***	0.26***	0.48***	0.74***	0.83***	-	0.70***	0.29***	0.28***	0.31***
11.ADES_ABS	0.20***	0.16***	0.32***	0.15**	0.13**	0.24***	0.37***	0.77***	0.74***	0.70***	-	0.17***	0.21***	0.25***
12.EDI_DT	−0.01	−0.02	0.12**	0.02	0.01	0.15**	0.36***	0.16*	0.24***	0.29***	0.17*	-	0.56***	0.72***
13.EDI_B	0.12*	0.09	0.26***	0.15**	0.06	0.11*	0.32***	0.23***	0.30***	0.28***	0.21***	0.56***	-	0.53***
14.EDI_BD	0.06	0.00	0.21***	0.11*	0.01	0.11*	0.40***	0.25***	0.28***	0.31***	0.25***	0.72***	0.53***	-

*ADES_ABS* Absorption, *ADES_AM* Amnesia, *ADES_DEP* Depersonalization, *ADES_PI* Passive Influence, *CTQ_EA* CTQ Emotional Abuse, *CTQ_EN* CTQ Emotional Neglect, *CTQ_PA* CTQ Physical Abuse, *CTQ_PN* CTQ Physical Neglect, *CTQ_SA* CTQ Sexual Abuse, *EDI_B* EDI-3 Bulimia, *EDI_BD* EDI-3 Body Dissatisfaction, *EDI_DT* EDI-3 Drive for Thinness, *PNI_GRD* B-PNI Grandiose Narcissism, *PNI_VLN* B-PNI Vulnerable Narcissism. *p <.05, **p <.01, ***p <.001

### Path Analysis

**Fig. 1:**
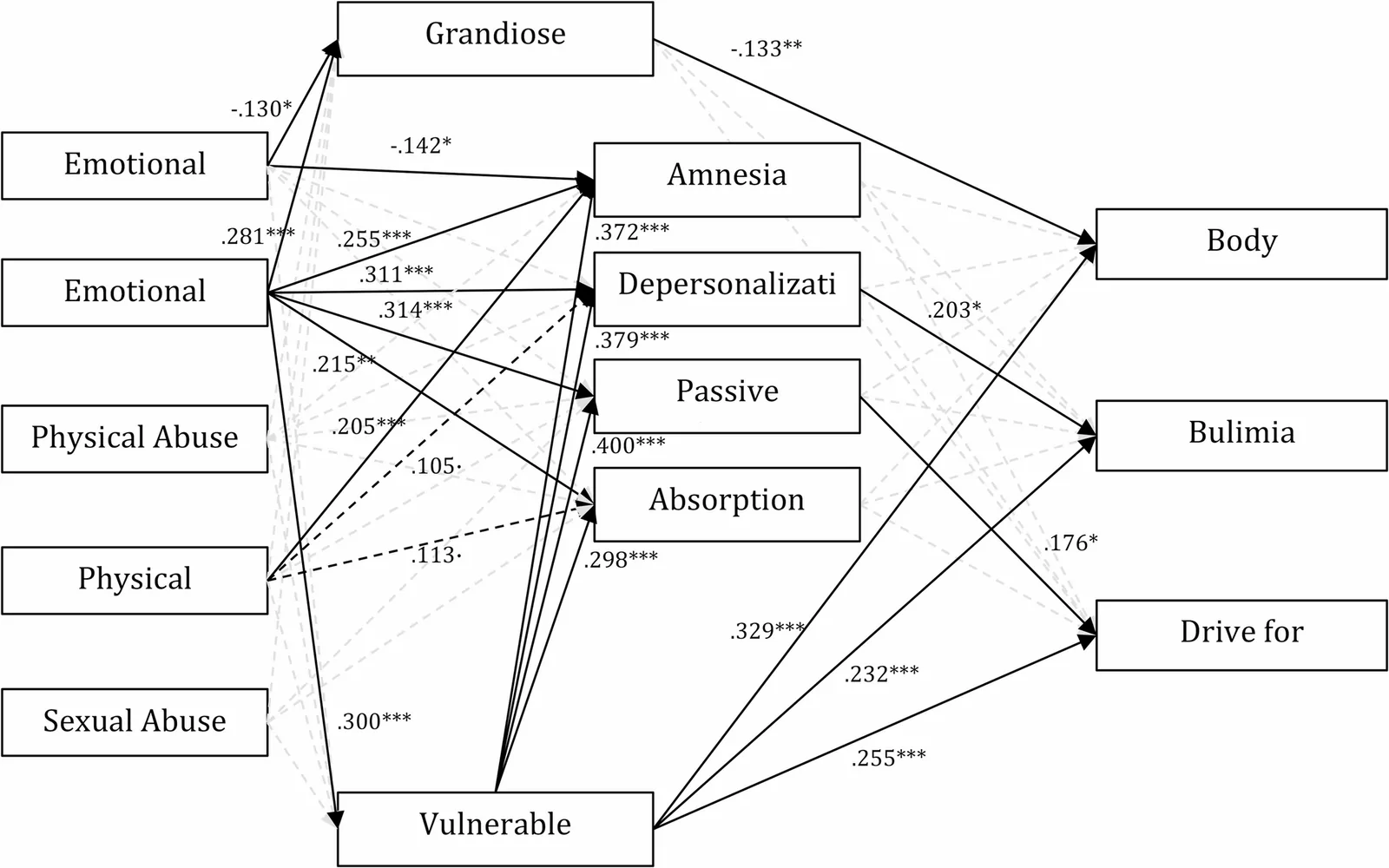
Path Analysis Model in the Total Sample. *Note.* Path analysis model in the total sample showing regression coefficients for the main variables. ***p < .001; **p< .01; *p < .05; ·*p* < .10. Dotted lines indicate effects at *p* < .10; faded dotted lines indicate nonsignificant effects

Model indices showed a good fit to the data: χ²_(17)_ = 30.68, *p* =.022; CFI = 0.99; TLI = 0.97; RMSEA = 0.04; SRMR = 0.02. The second model including a direct pathway of childhood maltreatment also displayed an adequate fit: χ²_(2)_ = 4.187, *p* =.123; CFI = 0.99; TLI = 0.96; RMSEA = 0.05; SRMR = 0.02. The ANOVA comparison of the two models revealed a significant difference χ²_diff(15)_ = 26.49, *p* =.033. The first model, not including a direct pathway from childhood maltreatment to the outcome variables, showed a better fit to the data (AIC = 29753; BIC = 30304; RMSEA = 0.04) compared to the second one, which instead included such direct pathway (AIC = 29756; BIC = 30396; RMSEA = 0.05). Moreover, in the second model, despite being tested, none of the direct effects between all types of childhood maltreatment and all the considered outcomes reached the significance threshold, supporting the retained indirect-effects model. The first model was therefore retained. The alternative model testing inverse relationships between variables also achieved a good fit χ²(17) = 35.14, *p* =.006; CFI = 0.99; TLI = 0.96; RMSEA = 0.05; SRMR = 0.02. When compared to the main model, marginal differences in the AIC and BIC indices were observed in favour of the latter (main model: AIC = 29753; BIC = 30304; inverse model: AIC = 29760; BIC = 30311). Several direct effects reached statistical significance. It is noteworthy that among all the types of childhood trauma, only emotional abuse consistently showed a significant direct relationship with all narcissism and dissociation sub-scales (see supplementary materials). Contrary to expectations, emotional neglect was negatively associated with grandiose narcissism (β=−0.13, *p* =.037, ci.lb.=−0.048, ci.up.=−0.002) and dissociative amnesia (β=−0.142, *p* =.024, ci.lb.=−0.124, ci.up.=−0.007). Grandiose narcissism in turn was not associated with any further variable except for body dissatisfaction, displaying a negative association (β=−0.133, *p* =.009, ci.lb.=−2.675, ci.up.=−0.373). Vulnerable narcissism was linked significantly to all dissociation and eating disorder sub-scales (see supplementary materials). Regression analyses revealed that only two facets of dissociation were significantly associated with eating disorder risk. Depersonalisation had a significant positive link to the bulimia sub-scale of the EDI-3 (β = 0.203, *p* =.019, ci.lb.=0.094, ci.up.=1.123), whereas passive influence had a significant positive link to the drive for thinness sub-scale (β = 0.176, *p* =.045, ci.lb.=0.023, ci.up.=1.257). Dissociative amnesia and absorption did not exhibit statistically significant associations with any of the EDI-3 sub-scales. The retained path analysis model for the total sample is presented in Fig. 1.

When examining the total indirect paths, grandiose narcissism was an intermediate variable in the relationship between childhood traumatic experiences (irrespective of the type) and bulimia through depersonalisation. The relationship between drive for thinness and all types of childhood trauma was statistically accounted for by vulnerable narcissism through depersonalisation and passive influence. The relationship between bulimia and all types of childhood trauma was instead accounted for by vulnerable narcissism through depersonalisation passive influence, and amnesia. Finally, the relationship between all types of traumata and body dissatisfaction was indirect and involved amnesia, passive influence and absorption but not depersonalisation. The standardised regression weights of the significant indirect effects summarised above are shown in the supplementary materials. When only paths consisting of individually significant regressions are examined, only emotional abuse shows two significant indirect associative links, which involve vulnerable narcissism, and account for drive for thinness via passive influence (β = 0.88; *p <*.001; ci.lb = 0.91; ci.ub.=2.25) and for bulimia via depersonalisation (β = 0.88; *p <*.001; ci.lb.=0.85; ci.ub.=1.99).

### Multi-group Analysis

**Fig. 2:**
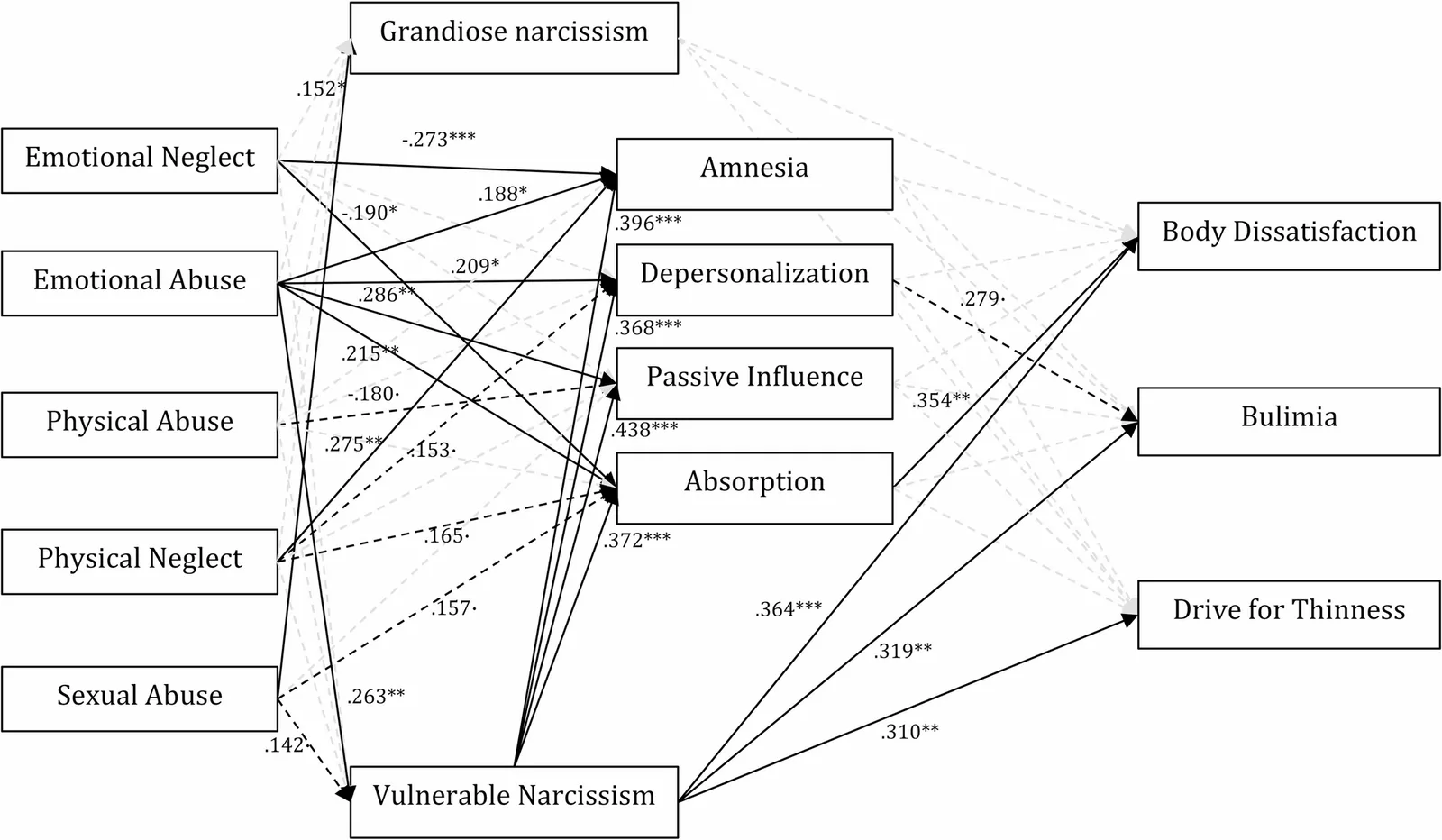
Path Analysis Model in the Male Subsample. *Note.* Path analysis model in the male subsample showing regression coefficients for the main variables. ***p < .001; **p< .01; *p < .05; ·*p *< .10. Dotted lines indicate effects at *p* < .10; faded dotted lines indicate nonsignificant effects

A multi-group analysis was carried out with gender as a grouping variable, and the chi-square difference test yielded a significant result (χ²_diff(58)_ = 82.247, *p* =.019), which indicated that the hypothesised model was not invariant across males and females. The models, estimated separately for the two groups, provided a good fit to the data. Males: χ²_(15)_ = 18.69, *p* =.23; CFI = 0.99; TLI = 0.98; RMSEA = 0.03; SRMR = 0.02. Females: χ²_(15)_ = 27.48, *p* =.025; CFI = 0.99; TLI = 0.95; RMSEA = 0.06; SRMR = 0.02. The path analysis model for the male subsample is presented in Fig. 2.

In the male group, a different pattern in the associations of the dissociation sub-scales was obtained, as the only significant direct link found was that of absorption to body dissatisfaction (β = 0.354, *p* =.002, ci.lb.=0.605, ci.up.=2.793). For both the males’ and females’ models the direct associations among variables other than the dissociation sub-scales kept the same general trend as in the complete model, even though some did not reach statistical significance. The only significant paths involving grandiose narcissism linked all types of abuse and neglect to body dissatisfaction with absorption as an intermediate link. Vulnerable narcissism accounted for the association between all childhood traumatic experiences and drive for thinness together with amnesia, passive influence, and absorption. The relationship between all types of traumata and bulimia was accounted for by vulnerable narcissism through amnesia, passive influence, and depersonalisation. Finally, childhood maltreatment was indirectly linked to body dissatisfaction with the intermediate role of vulnerable narcissism in combination with passive influence and absorption (see supplementary materials). The only complete indirect pathways with all significant or near significant regression coefficients were the ones connecting emotional abuse and vulnerable narcissism to body dissatisfaction via absorption (β = 0.82; *p =*.002; ci.lb.=0.49; ci.ub.=2.25) and to bulimia via depersonalisation (β = 0.91; *p =*.001; ci.lb.=0.58; ci.ub.=2.40).

**Fig. 3:**
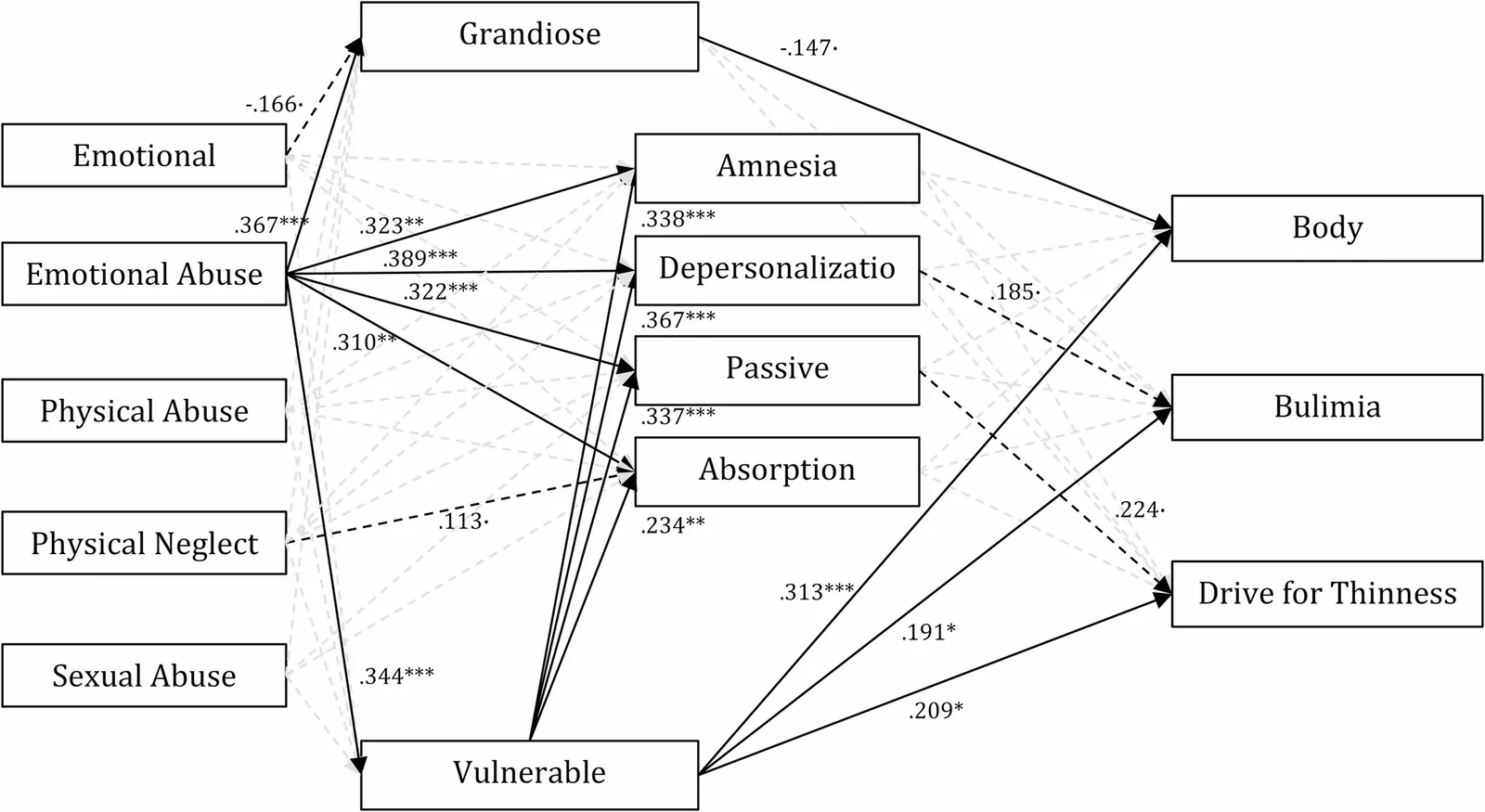
Path Analysis Model in the Female Subsample. *Note.* Path analysis model in the female subsample showing regression coefficients for the main variables. ***p < .001;**p < .01; *p < .05; ·*p* < .10. Dotted lines indicate effects at *p* < .10; faded dotted lines indicate nonsignificant effects

In the female group (Fig. 3) no complete path involved grandiose narcissism. The relationship between drive for thinness, bulimia and body dissatisfaction as endogenous variables and all types of childhood trauma as exogenous variables was characterized by indirect pathways through vulnerable narcissism only through depersonalisation and passive influence. Among the other two ADES subscales, amnesia, in combination with vulnerable narcissism, only functioned as a connector in the paths between emotional and physical abuse and body dissatisfaction. The only pathways composed by significant or near significant regression coefficients were the ones connecting emotional abuse, with the involvement of vulnerable narcissism, to drive for thinness via passive influence (β = 0.91; *p <*.001; ci.lb.=0.74; ci.ub.=2.65) and to bulimia via depersonalisation (β = 0.89; *p <*.001; ci.lb.=0.68; ci.ub.=2.23).

## Discussion

The present study examined whether narcissistic traits and dissociative experiences accounted for the indirect shared variance between childhood trauma and body dissatisfaction, drive for thinness, and bulimia in adolescents. The findings indicated that emotional abuse was the trauma dimension most consistently associated with narcissistic and dissociative variables, and that its associations with drive for thinness and bulimia were indirect. These results are consistent with previous studies highlighting areas of overlap between narcissism, dissociation, and eating-disorder-related features, but also provide a more specific account of the pathways linking these constructs in adolescence (Basten & Touyz, 2020; Bertele et al., 2022; Cipriano et al., 2024; Petrucelli, 2016). More specifically, the model suggests that narcissistic vulnerability may represent a form of self-organisation fragility associated with early traumatic experiences, particularly emotional abuse, and that this vulnerability may be linked to eating-disorder-related features through specific dissociative dimensions. Depersonalisation was mainly associated with bulimia, whereas passive influence was mainly associated with drive for thinness. Considering the model as a whole, divergent pathways stemming from childhood trauma can be identified. Grandiose narcissism showed a negative association with body dissatisfaction and was not positively associated with the eating-disorder-related outcomes, whereas vulnerable narcissism was positively associated with dissociative experiences, drive for thinness, and bulimia. These findings may reflect different psychological responses to traumatic threats to the self, with distinct profiles of vulnerability and symptom expression (Bonanno & Mancini, 2012; Sumner et al., 2021). Because the design was cross-sectional, these pathways should be interpreted as theoretically specified associations rather than causal developmental sequences.

With regard to a more detailed discussion of the individual components of the model, the first point to be examined is the prominent role played by emotional abuse, which among other types of adverse childhood experiences was the only one to clearly, albeit indirectly, establish a link to drive for thinness, bulimia, and body dissatisfaction. This result is in line with research indicating that emotional abuse constitutes a type of maltreatment with severe psychological sequelae (Dye, 2019; Montoro et al., 2022) and that it may hold a distinct place among other forms of trauma as a risk factor for body image and eating disturbances (Barakat et al., 2023). It should however be noted that whereas some studies confirm a similar specificity of emotional abuse (Monteleone et al., 2022), others report non-specific effects of all forms of childhood maltreatment in increasing eating and body related symptoms (Rabito-Alcón et al. 2020a; Vanderlinden et al. 1993). Bulimic tendencies can be more prevalent among adolescents with a history of sexual abuse (Robertson et al., 2013; Shenk & Fruzzetti, 2014), and childhood neglect has been linked to the inability to regulate intense emotions, which is a crucial predisposing factor for body dissatisfaction or dysfunctional dispositions towards food (Braden et al., 2021; Akhator-Eneka, 2024). Furthermore, a meta-analysis on the relationship between childhood neglect and disordered eating found a higher prevalence of emotional and physical neglect in individuals with body image disturbances and eating psychopathology (Izydorczyk, 2021; Kong & Bernstein, 2009). These inconsistencies could be explained by hypothesising that different traumata may influence body image and bulimic tendencies through diverse mechanisms and psychological pathways (Holman, 2012; Zajenkowski et al., 2018). The identification of the role of other adverse life events may become clearer with the addition of other factors which have not been considered in this study, such as beliefs regarding eating behaviours and emotions (Braden et al., 2021; Krause et al., 2003; Strodl & Wylie, 2020).

The connection of emotional abuse to all PNI and ADES sub-scales also suggests that this type of trauma may play a more significant role in the development of narcissistic and dissociative features as well compared to other kinds of maltreatment (Foster et al., 2003; Pincus et al., 2014). The importance of vulnerable narcissism in the present model is consistent with existing literature that highlights the role of feelings of inadequacy, hypersensitivity to criticism, and unstable self-esteem as important risk factors for developing harmful attitudes towards one’s body (Caiozzo et al., 2018; Canale et al., 2022; Gee, 2020). Conversely, grandiose narcissism was not significantly associated with any eating disorder-related variable except for a negative effect on body dissatisfaction. This particular result implies that, as noted by other authors (Carrotte & Anderson, 2019), the protective self-view of grandiose narcissism may serve as a buffer against body image concerns. The negative link of emotional neglect to grandiose narcissism found in the total sample partially contrasts with previous indications of a positive association between experiences of neglect during childhood and the display of grandiosity traits in adulthood (Gao et al., 2024). However, the combination of a positive association with emotional abuse and a negative association with emotional neglect in the present study suggests that involved and non-negligent but at the same time abusive early relationships were more likely to contribute to narcissistic grandiosity. Repeated overvaluation by one’s parents, which would be consistent with low self-reported emotional neglect, has been proposed as promoting heightened grandiose traits. In fact, overvaluing could relay the implicit message that, if the idealised image of the child held by the parents would not hold true, rejection would then ensue, triggering self-inflating compensatory mechanisms (Benjamin, 2002; Otway & Vignoles, 2006; Imamoglu & Durak Batigun, 2020).

The present findings should also be interpreted in relation to previous work reporting different patterns of association between narcissism and dissociation. For example, Şar and Türk-Kurtça (2021) described traumatic narcissism and dissociative depression as closely connected clinical phenomena, suggesting that both grandiose and vulnerable narcissistic configurations may be relevant to dissociative processes. Our results do not contradict this broader view. Rather, they suggest that, in this non-clinical adolescent sample, vulnerable narcissism was more strongly related to dissociative experiences and eating-disorder-related features than grandiose narcissism. Differences between studies may reflect differences in sample type, developmental stage, symptom severity, and the instruments used to assess narcissistic traits (Bertele et al., 2022; Miller et al., 2017; Pincus et al., 2009; Şar & Türk-Kurtça, 2021).

A negative link between emotional neglect and dissociative amnesia was also found. As with grandiose narcissism, it is possible that in our sample emotionally available but overbearing, intrusive or abusive parents and family members were involved in a stronger disorganization of autobiographical memory rather than emotionally absent or unavailable ones. It has also been advocated that trauma alone can be insufficient to explain dissociative phenomena and that other determinants of dissociation should also be considered in both clinical practice and research (Dell, 2023; Lynn et al., 2012). Mediators or moderators that were absent from the model, or an interaction between the variables considered could further condition the relationship between trauma and dissociative amnesia.

Our findings also indicate that different facets of dissociation, such as depersonalisation and passive influence, may play distinct roles in statistically accounting for body and eating related disturbances. Depersonalisation was mainly linked to bulimic tendencies, while passive influence had a more selective relationship with drive for thinness. Feelings of depersonalisation and derealisation, together with a quality of involuntariness, have been posited to be characteristic of eating pathology, particularly of bulimia (La Mela et al., 2010). Such observation adds to previous research indicating that dissociation is linked to the severity of binge eating (Belli et al., 2019; Palmisano et al., 2018), and that higher dissociation scores can be found in the binge-purging subtype of anorexia nervosa relative to the restrictive subtype (Palmisano et al., 2018). Detachment and a sense of estrangement from one’s body and mental activity, i.e. depersonalization, which signal the dissolution of higher integrative levels of mental functioning (Farina & Meares, 2022), could be seen as a predisposing condition to the loss of control over bodily impulses, i.e. bulimic tendencies. This reading follows the neo-Jacksonian principle of a dynamic combination of negative and positive processes in symptom formation (Farina et al., 2005; Meares, 1999). In other words, the loss of higher regulatory functions (negative processes) can result in unrestrained actions facilitated by lower levels of mental functioning (positive processes), which would be correlative to the subjective experience of a disconnection/extraneity from oneself. The association of passive influence with drive for thinness, on the other hand, may suggest that a dissociative feeling of altered agency and volition can prompt attempts to manipulate the body’s appearance and needs as a way of regaining control over one’s psychological activity. Such tentative explanation parallels other theoretical models according to which disordered eating can be employed to maladaptively modulate negative and overwhelming emotional states (Cella et al., 2022; Haynos & Fruzzetti, 2011). In keeping with this perspective, albeit with regards to a different form of trauma, it has been proposed that as childhood sexual abuse brings forth emotional dysregulation and overpowering feelings of a lack of control, it can in turn fuel a need to regain power through restrictive eating behaviours (Rabito-Alcón et al. 2020b; Smolak and Murnen 2002).

The most notable difference between male and female participants concerned the role of absorption. Absorption is a debated construct because it has often been described as a disposition toward intense imaginative involvement, daydreaming, and changes in attention, but it has also been linked to clinically relevant dissociative and psychopathological phenomena (Kihlstrom, 2005; Lyssenko et al., 2018; Soffer-Dudek et al., 2015). In the present study, absorption was involved in the pathway between childhood trauma and body dissatisfaction only among males. This finding may suggest that, in male adolescents, trauma-related and narcissistic vulnerability can be connected to body dissatisfaction through internally focused imaginative or self-referential states. This interpretation is consistent with evidence linking narcissistic traits to body dissatisfaction and body-image concerns (Carrotte & Anderson, 2019; Purton et al., 2018). It is also compatible with recent work on internal moderation, a construct describing internal self-regulatory processes related to dissociation and absorption (Ayas et al., 2025). However, internal moderation was not measured in the present study. Future research should directly assess this construct to clarify whether absorption contributes to eating-disorder-related symptoms through altered internal self-regulation.

It should be clearly stated that the account provided thus far for the interpretation of dissociative phenomena in the present model assumes that dissociation is a specific kind of disintegrative mental process set off by traumatic events. An alternative account can however be provided if dissociation is considered as a defence against trauma, rather than as a mere consequence (Dell, 2023). Dissociation may function as a *purposeful* secondary trauma response, enabling individuals to freeze, avoid, or detach from traumatic experiences (Rabito-Alcón et al. 2020b; Smolak and Murnen 2002). Following this line of reasoning, it could be posited that some traumatised individuals may learn to dissociate from their emotions to cope with traumatic experiences, and that they may consequently adopt harmful attitudes towards their body as a means of avoiding trauma-related feelings and memories (Beato Fernández & Rodríguez Cano, 2003; Rodríguez-Cano et al., 2012; Campbell et al., 2009; Safer et al., 2009; Duarte et al., 2017). The standing of these different explanations depends upon current controversies in the study of dissociation and on the various definitions of such construct, with a major divide being the conceptualisation of dissociation as either an effect of insufficient synthetic/integrative processes or as an evasive defence strategy akin to an extreme form of avoidance against a threat which is inescapable by other means (Van der Hart & Dorahy, 2023). Our results do not allow us to clarify the role of dissociation more precisely.

Overall, the present study provides evidence of specific associations among childhood trauma, narcissistic traits, dissociative experiences, and eating-disorder-related features in adolescents. The findings suggest that emotional abuse may be particularly relevant to narcissistic vulnerability and dissociative experiences, and that different dissociative dimensions may be associated with different eating-related outcomes. Depersonalisation was mainly associated with bulimic tendencies, passive influence was mainly associated with drive for thinness, and absorption was involved in the association with body dissatisfaction among males. These results support the importance of examining dissociation as a multidimensional construct rather than as a single global variable (Briere et al., 2005; Ellickson-Larew et al., 2020; Quattropani et al., 2022). Clinically, the findings suggest that trauma-exposed adolescents with eating-disorder-related symptoms may benefit from careful assessment of dissociative experiences and narcissistic vulnerability. However, because the study was cross-sectional and based on self-report measures, the results should be interpreted cautiously and require replication in longitudinal and clinical samples.

### Limitations and Future Directions

The cross-sectional design of this investigation prevents any causal interpretation of the reported associations. Although the path model was ordered according to theoretical and developmental assumptions, the data do not demonstrate that childhood trauma caused narcissistic traits or dissociation, or that narcissistic traits and dissociation caused eating-disorder-related outcomes. While an alternative, inverse model was also tested and showed a marginally worse fit to the data, the difference was minimal. More to the point, design limitations with regards to causal relationships between variables cannot be entirely offset by the analytical strategy (Maxwell & Cole, 2007; O’Laughlin et al., 2018). In fact other authors have argued in favour of different causal chains linking the variables explored here (e.g. Talmon & Ginzburg, 2019), and future studies adopting a longitudinal methodology are necessary to verify and refine competing explanatory models. New approaches more suitable for cross-sectional mediation analyses should also be considered (Weems, 2025). In order to verify the directionality of the observed pathways and to document the temporal sequence between trauma, narcissistic traits, dissociative experiences, and eating or body image disturbances, longitudinal research would be required (Becker et al., 2019). Future studies should also test alternative models, including reciprocal, interconnected, and network models, because the variables examined in this study may influence one another over time. Self-report measures also present some challenges, notably in the context of dissociation assessment, as the reliability of self-reported dissociative symptoms may be restricted by the phenomenon itself (Akyüz et al., 1999). Internal consistency was acceptable for most subscales. However, Cronbach’s alpha was below 0.70 for the CTQ-SF Physical Neglect subscale (α = 0.60) and the A-DES Absorption subscale (α = 0.68), although their corresponding McDonald’s omega coefficients were acceptable (ω = 0.71 and 0.75, respectively). Although McDonald’s omega has been recommended as a reliability estimate that depends on less restrictive assumptions than Cronbach’s alpha (Hayes & Coutts, 2020), the acceptable omega values do not completely remove concerns about measurement precision. Associations involving Physical Neglect and Absorption should therefore be interpreted cautiously. Lower internal consistency has also been reported for the CTQ-SF Physical Neglect subscale and the A-DES Absorption subscale in previous psychometric research (De Pasquale et al., 2016; Georgieva et al., 2021). Future studies should replicate these findings using measures with stronger and more stable subscale reliability. In addition, although the measures used in this study have been applied to adolescent samples, the use of retrospective trauma questionnaires and self-report symptom scales in younger adolescents requires caution because age, comprehension level, and response style may affect the accuracy of reporting (Armstrong et al., 1997; De Pasquale et al., 2016; Hagborg et al., 2022). The amnestic features of dissociation could prevent the person from freely accessing the self-relevant information that is collected by questionnaires addressing past trauma or other symptomatic experiences. The disruption of consciousness and the dysintegration of psychological processes could make it challenging for affected individuals to accurately observe and report on their own mental states. To verify self-reported data and acquire a deeper comprehension of dissociative phenomena, future research should contemplate the implementation of multimodal assessment methods, including clinical interviews, behavioural observations, and neuropsychological tests. In addition, this model fails to completely elucidate the mechanisms by which post-traumatic vulnerability characterised by dissociative features specifically contributes to body related symptomatology. Future research should concentrate on factors that contribute to the development of eating disorder symptoms, including disruptions in the mind-body connection, identity development, and interpersonal difficulties (Merwin et al., 2010). Investigating these factors may help clarify why certain trauma survivors develop body image concerns while others do not. A more detailed examination of different subtypes of dissociation (e.g., depersonalisation, derealisation, and identity fragmentation) and their specific impacts on eating behaviours would also be beneficial along with investigation of the co-occurrence of other personality vulnerabilities, such as borderline or avoidant traits, which have been linked to both dissociation and eating disorder related features (Sansone & Sansone, 2011).

Additionally, the study’s participants consisted of high school students from southern Italy, which may limit the generalisability of the findings to other age groups, clinical populations, and cultural contexts. Previous research has shown that the expression of trauma-related symptoms, including dissociative and narcissistic traits, can vary significantly across cultures, as cultural norms and values influence how individuals process and express psychological distress (Montoro et al., 2022). Replicating this model in diverse populations, including clinical samples with more severe symptomatology, would help determine its broader applicability. Another limitation is the study’s concentration on subclinical individuals, which may inadequately represent the intricate manifestations of dissociation and narcissism found in clinical environments. Finally, developmental considerations, such as the timing, duration, and nature of childhood trauma, were not directly examined in our study. The developmental stage at which trauma occurs can have a profound impact on psychological outcomes, with earlier traumas potentially leading to more pervasive personality disruptions (Van der Kolk, 2005). Longitudinal research tracking trauma exposure, dissociative symptoms, narcissistic traits, and eating-disorder-related features from childhood to adolescence and adulthood would be useful for identifying critical periods, developmental pathways, and possible reciprocal effects among these constructs.

## Conclusions

This study suggests that emotional abuse, vulnerable narcissism, and specific dissociative dimensions are associated with drive for thinness and bulimic tendencies in adolescents. The findings indicate that the associations between childhood trauma and eating-disorder-related features may be indirect and may involve narcissistic vulnerability, depersonalisation, and passive influence. Gender differences also emerged, with absorption being involved in the association between childhood trauma and body dissatisfaction among males but not females. These results support the importance of examining intermediary psychological factors when studying eating-disorder-related symptoms in trauma-exposed adolescents (Basten & Touyz, 2020; Bertele et al., 2022; Cipriano et al., 2024; Quattropani et al., 2022). From a clinical perspective, assessment of trauma-related symptoms in adolescents with body image or eating-related problems may benefit from including dissociative experiences and narcissistic vulnerability. Because the study was cross-sectional and based on self-report measures, the findings should be interpreted with caution and require replication in longitudinal and clinical samples.

## Supplementary Information

Below is the link to the electronic supplementary material.

Supplementary Material 1 (DOCX 50.9 KB)

## Data Availability

No datasets were generated or analysed during the current study.

## Declarations

### Artificial Intelligence Statement

No generative artificial intelligence tools were used in the conduct of the research or in the preparation of the scientific content of this manuscript. Standard spelling and grammar-checking software was used solely for proofreading and language correction. All revisions were reviewed and approved by the authors, who take full responsibility for the final manuscript.
