# Supplementary material for: The Role of Narcissism and Dissociation in the Indirect Relationship Between Childhood Trauma and Body Dissatisfaction, Bulimia, and Drive for Thinness in a Sample of Adolescents: A Path Analysis Model
Source: J Child Adolesc Trauma. 2026 Aug 8;19(4):2827–44. doi: 10.1007/s40653-026-00958-4 (PMC13737212; doi:10.1007/s40653-026-00958-4)
Supplement: Supplementary file 1 [file 40653_2026_958_MOESM1_ESM.docx]

Supplementary Table S1. Significant indirect effects in the total sample. Effects mediated by amnesia are marked in green, those mediated by passive influence are marked in red, those mediated by depersonalization in blue, and those mediated by absorption in yellow. EA = Emotional Abuse; PA = Physical Abuse; EN = Emotional Neglect; PN = Physical Neglect; SA = Sexual Abuse; VLN = Vulnerable Narcissism; GRD = Grandiose Narcissism; AMN = Amnesia; DEP = Depersonalization; PI = Passive Influence; ABS = Absorption; DT = Desire for Thinness; B = Bulimia; BD = Body Dissatisfaction.

|  | β | *p* | ci.lower | ci.upper |
| --- | --- | --- | --- | --- |
| Effects on **drive for thinness** |  |  |  |  |
| via vulnerable narcissism |  |  |  |  |
| EA > VLN > PI > DT | 0.88 | <.001 | 0.911 | 2.246 |
| EA > VLN > DEP > DT | 0.74 | .008 | 0.281 | 1.867 |
| EN > VLN > PI > DT | 0.57 | <.001 | 0.839 | 2.168 |
| EN > VLN > DEP > DT | 0.43 | .013 | 0.207 | 1.787 |
| PA > VLN > PI > DT | 0.63 | <.001 | 0.857 | 2.168 |
| PA > VLN > DEP > DT | 0.49 | .012 | 0.219 | 1.787 |
| PN > VLN > PI > DT | 0.49 | <.001 | 0.805 | 2.192 |
| PN > VLN > DEP > DT | 0.35 | .017 | 0.171 | 1.802 |
| SA > VLN > PI > DT | 0.64 | <.001 | 0.869 | 2.142 |
| SA > VLN > DEP > DT | 0.50 | .011 | 0.236 | 1.802 |
| Effects on **bulimia** |  |  |  |  |
| via grandiose narcissism |  |  |  |  |
| EA > GRD > DEP > B | 0.497 | .018 | 0.118 | 1.277 |
| EN > GRD > DEP > B | 0.085 | .037 | 0.033 | 1.202 |
| PA > GRD > DEP > B | 0.208 | .032 | 0.048 | 1.227 |
| PN > GRD > DEP > B | 0.121 | .040 | 0.036 | 1.198 |
| SA > GRD > DEP > B | 0.281 | .026 | 0.078 | 1.244 |
| via vulnerable narcissism |  |  |  |  |
| EA > VLN > AMN > B | 0.684 | .002 | 0.318 | 1.449 |
| EA > VLN > PI > B | 0.709 | .001 | 0.422 | 1.534 |
| EA > VLN > DEP > B | 0.883 | <.001 | 0.850 | 1.995 |
| EA > VLN > ABS > B | 0.567 | .033 | 0.025 | 1.005 |
| EN > VLN > AMN > B | 0.377 | .006 | 0.246 | 1.367 |
| EN > VLN > PI > B | 0.402 | .002 | 0.346 | 1.456 |
| EN > VLN > DEP > B | 0.575 | <.001 | 0.771 | 1.910 |
| PA > VLN > AMN > B | 0.436 | .005 | 0.259 | 1.389 |
| PA > VLN > PI > B | 0.461 | .001 | 0.369 | 1.475 |
| PA > VLN > DEP > B | 0.634 | <.001 | 0.789 | 1.932 |
| PN > VLN > AMN > B | 0.294 | .008 | 0.215 | 1.336 |
| PN > VLN > PI > B | 0.319 | .003 | 0.311 | 1.422 |
| PN > VLN > DEP > B | 0.493 | <.001 | 0.745 | 1.883 |
| SA > VLN > AMN > B | 0.443 | .005 | 0.266 | 1.399 |
| SA > VLN > PI > B | 0.468 | .001 | 0.368 | 1.477 |
| SA > VLN > DEP > B | 0.641 | <.001 | 0.794 | 1.934 |
| Effects on **body dissatisfaction** |  |  |  |  |
| via vulnerable narcissism |  |  |  |  |
| EA > VLN > AMN > BD | 0.697 | .007 | 0.242 | 1.622 |
| EA > VLN > PI > BD | 0.776 | .001 | 0.561 | 1.986 |
| EA > VLN > ABS > BD | 0.658 | .019 | 0.130 | 1.691 |
| EN > VLN > AMN > BD | 0.390 | .013 | 0.177 | 1.548 |
| EN > VLN > PI > BD | 0.468 | .001 | 0.488 | 1.911 |
| EN > VLN > ABS > BD | 0.351 | .032 | 0.055 | 1.619 |
| PA > VLN > AMN > BD | 0.449 | .012 | 0.187 | 1.560 |
| PA > VLN > PI > BD | 0.528 | .001 | 0.502 | 1.930 |
| PA > VLN > ABS > BD | 0.410 | .028 | 0.084 | 1.629 |
| PN > VLN > AMN > BD | 0.307 | .017 | 0.136 | 1.505 |
| PN > VLN > PI > BD | 0.386 | .002 | 0.448 | 1.881 |
| PN > VLN > ABS > BD | 0.268 | .038 | 0.033 | 1.579 |
| SA > VLN > AMN > BD | 0.456 | .011 | 0.193 | 1.568 |
| SA > VLN > PI > BD | 0.535 | .001 | 0.510 | 1.933 |
| SA > VLN > ABS > BD | 0.417 | .028 | 0.075 | 1.640 |

Supplementary Table S2. Significant indirect effects in the male subsample.

|  | β | *p* | ci.lower | ci.upper |
| --- | --- | --- | --- | --- |
| Effects on **drive for thinness** |  |  |  |  |
| via vulnerable narcissism |  |  |  |  |
| EA > VLN > AMN > DT | 0.667 | .026 | 0.089 | 1.621 |
| EA > VLN > PI > DT | 0.854 | .002 | 0.556 | 2.399 |
| EA > VLN > ABS > DT | 0.816 | .002 | 0.492 | 2.249 |
| EN > VLN > AMN > DT | 0.418 | .036 | 0.039 | 1.551 |
| EN > VLN > PI > DT | 0.606 | .003 | 0.497 | 2.340 |
| EN > VLN > ABS > DT | 0.567 | .003 | 0.443 | 2.186 |
| PA > VLN > AMN > DT | 0.377 | .042 | 0.009 | 1.542 |
| PA > VLN > PI > DT | 0.565 | .004 | 0.486 | 2.324 |
| PA > VLN > ABS > DT | 0.526 | .004 | 0.406 | 2.166 |
| PN > VLN > PI > DT | 0.499 | .004 | 0.463 | 2.299 |
| PN > VLN > ABS > DT | 0.461 | .004 | 0.402 | 2.152 |
| SA > VLN > AMN > DT | 0.546 | .030 | 0.070 | 1.597 |
| SA > VLN > PI > DT | 0.734 | .003 | 0.549 | 2.381 |
| SA > VLN > ABS > DT | 0.695 | .002 | 0.478 | 2.225 |
| Effects on **bulimia** |  |  |  |  |
| via vulnerable narcissism |  |  |  |  |
| EA > VLN > AMN > B | 0.705 | .007 | 0.263 | 1.673 |
| EA > VLN > PI > B | 0.667 | .034 | 0.108 | 1.730 |
| EA > VLN > DEP > B | 0.911 | .001 | 0.580 | 2.404 |
| EN > VLN > AMN > B | 0.457 | .012 | 0.207 | 1.595 |
| EN > VLN > PI > B | 0.419 | .050 | 0.045 | 1.670 |
| EN > VLN > DEP > B | 0.662 | .002 | 0.515 | 2.331 |
| PA > VLN > AMN > B | 0.416 | .013 | 0.194 | 1.586 |
| PA > VLN > PI > B | 0.378 | .053 | 0.040 | 1.647 |
| PA > VLN > DEP > B | 0.621 | .002 | 0.487 | 2.335 |
| PN > VLN > AMN > B | 0.350 | .016 | 0.162 | 1.564 |
| PN > VLN > PI > B | 0.312 | .065 | 0.003 | 1.642 |
| PN > VLN > DEP > B | 0.556 | .002 | 0.497 | 2.292 |
| SA > VLN > AMN > B | 0.585 | .009 | 0.234 | 1.652 |
| SA > VLN > PI > B | 0.547 | .038 | 0.096 | 1.702 |
| SA > VLN > DEP > B | 0.790 | .001 | 0.564 | 2.374 |
| Effects on **body dissatisfaction** |  |  |  |  |
| via grandiose narcissism |  |  |  |  |
| EA > GRD > ABS > BD | 0.541 | .003 | 0.603 | 2.945 |
| EN > GRD > ABS > BD | 0.263 | .005 | 0.539 | 2.878 |
| PA > GRD > ABS > BD | 0.319 | .005 | 0.525 | 2.900 |
| PN > GRD > ABS > BD | 0.301 | .005 | 0.525 | 2.888 |
| SA > GRD > ABS > BD | 0.530 | .003 | 0.604 | 2.951 |
| via vulnerable narcissism |  |  |  |  |
| EA > VLN > PI > BD | 0.785 | .013 | 0.304 | 2.375 |
| EA > VLN > ABS > BD | 0.989 | <.001 | 1.268 | 3.547 |
| EN > VLN > PI > BD | 0.537 | .018 | 0.232 | 2.305 |
| EN > VLN > ABS > BD | 0.741 | <.001 | 1.194 | 3.499 |
| PA > VLN > PI > BD | 0.496 | .018 | 0.236 | 2.297 |
| PA > VLN > ABS > BD | 0.700 | <.001 | 1.181 | 3.484 |
| PN > VLN > PI > BD | 0.430 | .021 | 0.201 | 2.269 |
| PN > VLN > ABS > BD | 0.634 | <.001 | 1.164 | 3.456 |
| SA > VLN > PI > BD | 0.664 | .014 | 0.279 | 2.336 |
| SA > VLN > ABS > BD | 0.868 | <.001 | 1.230 | 3.545 |

Supplementary Table S3. Significant indirect effects in the female subsample.

|  | β | *p* | ci.lower | ci.upper |
| --- | --- | --- | --- | --- |
| Effects on **drive for thinness** |  |  |  |  |
| via vulnerable narcissism |  |  |  |  |
| EA > VLN > PI > DT | 0.905 | <.001 | 0.741 | 2.653 |
| EA > VLN > DEP > DT | 0.867 | .014 | 0.305 | 2.709 |
| EN > VLN > PI > DT | 0.503 | .001 | 0.647 | 2.567 |
| EN > VLN > DEP > DT | 0.465 | .020 | 0.236 | 2.615 |
| PA > VLN > PI > DT | 0.672 | .001 | 0.693 | 2.621 |
| PA > VLN > DEP > DT | 0.634 | .016 | 0.276 | 2.672 |
| PN > VLN > PI > DT | 0.488 | .001 | 0.619 | 2.554 |
| PN > VLN > DEP > DT | 0.450 | .022 | 0.214 | 2.610 |
| SA > VLN > PI > DT | 0.564 | .001 | 0.654 | 2.575 |
| SA > VLN > DEP > DT | 0.526 | .018 | 0.251 | 2.621 |
| Effects on **bulimia** |  |  |  |  |
| via vulnerable narcissism |  |  |  |  |
| EA > VLN > PI > B | 0.706 | .021 | 0.120 | 1.725 |
| EA > VLN > DEP > B | 0.897 | <.001 | 0.675 | 2.226 |
| EN > VLN > PI > B | 0.303 | .035 | 0.031 | 1.627 |
| EN > VLN > DEP > B | 0.495 | .001 | 0.594 | 2.136 |
| PA > VLN > PI > B | 0.472 | .027 | 0.084 | 1.674 |
| PA > VLN > DEP > B | 0.664 | <.001 | 0.640 | 2.176 |
| PN > VLN > PI > B | 0.288 | .040 | 0.007 | 1.605 |
| PN > VLN > DEP > B | 0.480 | .001 | 0.568 | 2.115 |
| SA > VLN > PI > B | 0.365 | .033 | 0.048 | 1.641 |
| SA > VLN > DEP > B | 0.556 | .001 | 0.618 | 2.132 |
| Effects on **body dissatisfaction** |  |  |  |  |
| via vulnerable narcissism |  |  |  |  |
| EA > VLN > AMN > BD | 0.751 | .027 | 0.109 | 2.082 |
| EA > VLN > PI > BD | 0.795 | .009 | 0.308 | 2.270 |
| EA > VLN > DEP > BD | 0.847 | .017 | 0.286 | 2.677 |
| EN > VLN > AMN > BD | 0.348 | .043 | 0.021 | 1.995 |
| EN > VLN > PI > BD | 0.393 | .015 | 0.214 | 2.177 |
| EN > VLN > DEP > BD | 0.445 | .025 | 0.199 | 2.588 |
| PA > VLN > AMN > BD | 0.517 | .035 | 0.056 | 2.041 |
| PA > VLN > PI > BD | 0.561 | .012 | 0.274 | 2.226 |
| PA > VLN > DEP > BD | 0.614 | .020 | 0.251 | 2.636 |
| PN > VLN > PI > BD | 0.377 | .017 | 0.203 | 2.160 |
| PN > VLN > DEP > BD | 0.430 | .027 | 0.186 | 2.588 |
| SA > VLN > AMN > BD | 0.410 | .041 | 0.029 | 2.019 |
| SA > VLN > PI > BD | 0.454 | .015 | 0.231 | 2.191 |
| SA > VLN > DEP > BD | 0.506 | .023 | 0.213 | 2.603 |

Supplementary Table S4. Regression table for the total sample with significant and near-significant effects.

|  | β | *p* | ci.lower | ci.upper |
| --- | --- | --- | --- | --- |
| Grandiose Narcissism ~ |  |  |  |  |
| Emotional Abuse | 0.281 | <.001 | 0.032 | 0.080 |
| Emotional Neglect | -0.130 | .037 | -0.048 | -0.002 |
| Vulnerable Narcissism ~ |  |  |  |  |
| Emotional Abuse | 0.300 | <.001 | 0.045 | 0.104 |
| sex | 0.216 | <.001 | 0.257 | 0.642 |
| Amnesia ~ |  |  |  |  |
| Vulnerable Narcissism | 0.372 | <.001 | 0.529 | 0.986 |
| Physical Neglect | 0.205 | <.001 | 0.073 | 0.245 |
| Emotional Abuse | 0.255 | <.001 | 0.069 | 0.193 |
| Emotional Neglect | -0.142 | .024 | -0.124 | -0.007 |
| Passive Influence ~ |  |  |  |  |
| Vulnerable Narcissism | 0.372 | <.001 | 0.630 | 1.098 |
| Physical Neglect | 0.205 | <.001 | -0.030 | 0.146 |
| Emotional Abuse | 0.255 | <.001 | 0.099 | 0.238 |
| Emotional Neglect | -0.142 | .024 | -0.092 | 0.028 |
| Depersonalization ~ |  |  |  |  |
| Vulnerable Narcissism | 0.379 | <.001 | 0.527 | 0.938 |
| Physical Neglect | 0.105 | .057 | -0.002 | 0.161 |
| Emotional Abuse | 0.311 | <.001 | 0.093 | 0.210 |
| Absorption ~ |  |  |  |  |
| Vulnerable Narcissism | 0.298 | <.001 | 0.330 | 0.779 |
| Physical Neglect | 0.113 | .062 | -0.005 | 0.164 |
| Emotional Abuse | 0.215 | .002 | 0.037 | 0.162 |
| Desire for thinness ~ |  |  |  |  |
| Vulnerable Narcissism | 0.255 | <.001 | 1.050 | 2.981 |
| Passive Influence | 0.176 | .045 | 0.023 | 1.257 |
| sex | 0.243 | <.001 | 2.477 | 5.491 |
| Bulimia ~ |  |  |  |  |
| Vulnerable Narcissism | 0.156 | <.001 | 0.754 | 3.042 |
| Depersonalization | 0.203 | .019 | 0.019 | 1.123 |
| sex | 0.156 | .001 | 0.754 | 3.042 |
| Body Dissatisfaction ~ |  |  |  |  |
| Vulnerable Narcissism | 0.329 | <.001 | 1.979 | 4.057 |
| Grandiose Narcissism | -0.133 | .009 | -2.675 | -0.373 |
| sex | 0.277 | <.001 | 3.554 | 6.894 |

Supplementary Table S5. Regression table for the female subsample with significant and near-significant effects.

|  | β | *p* | ci.lower | ci.upper |
| --- | --- | --- | --- | --- |
| Grandiose Narcissism ~ |  |  |  |  |
| Emotional Abuse | 0.367 | <.001 | 0.039 | 0.098 |
| Emotional Neglect | -0.166 | .055 | -0.060 | 0.002 |
| Vulnerable Narcissism ~ |  |  |  |  |
| Emotional Abuse | 0.345 | <.001 | 0.037 | 0.117 |
| age | -0.175 | .006 | -0.175 | -0.029 |
| Amnesia ~ |  |  |  |  |
| Vulnerable Narcissism | 0.338 | <.001 | 0.409 | 1.080 |
| Emotional Abuse | 0.323 | .001 | 0.071 | 0.262 |
| Passive Influence ~ |  |  |  |  |
| Vulnerable Narcissism | 0.337 | <.001 | 0.450 | 1.147 |
| Emotional Abuse | 0.322 | .001 | 0.074 | 0.272 |
| Depersonalization ~ |  |  |  |  |
| Vulnerable Narcissism | 0.366 | <.001 | 0.472 | 1.001 |
| Emotional Abuse | 0.389 | <.001 | 0.096 | 0.269 |
| Absorption ~ |  |  |  |  |
| Vulnerable Narcissism | 0.233 | .006 | 0.142 | 0.821 |
| Emotional Abuse | 0.309 | .003 | 0.055 | 0.242 |
| Desire for thinness ~ |  |  |  |  |
| Vulnerable Narcissism | 0.210 | .018 | 0.362 | 3.370 |
| Passive Influence | 0.223 | .075 | -0.120 | 1.742 |
| Bulimia ~ |  |  |  |  |
| Vulnerable Narcissism | 0.191 | .029 | 0.115 | 2.425 |
| Depersonalization | 0.186 | .094 | -0.100 | 1.351 |
| Body Dissatisfaction ~ |  |  |  |  |
| Vulnerable Narcissism | 0.314 | <.001 | 1.321 | 4.404 |
| Grandiose Narcissism | -0.147 | .065 | -3.415 | 0.076 |

Supplementary Table S6. Regression table for the male subsample with significant and near-significant effects.

|  | β | *p* | ci.lower | ci.upper |
| --- | --- | --- | --- | --- |
| Grandiose Narcissism ~ |  |  |  |  |
| Emotional Abuse | 0.163 | .096 | -0.008 | 0.077 |
| Sexual Abuse | 0.152 | .052 | -0.002 | 0.081 |
| Vulnerable Narcissism ~ |  |  |  |  |
| Emotional Abuse | 0.345 | <.001 | 0.037 | 0.117 |
| Sexual Abuse | 0.142 | 0.078 | -0.010 | 0.095 |
| Amnesia ~ |  |  |  |  |
| Vulnerable Narcissism | 0.396 | <.001 | 0.483 | 1.095 |
| Physical Neglect | 0.275 | .001 | 0.082 | 0.301 |
| Emotional Abuse | 0.323 | .001 | 0.071 | 0.262 |
| Emotional Neglect | -0.275 | <.001 | -0.192 | -0.058 |
| Passive Influence ~ |  |  |  |  |
| Vulnerable Narcissism | 0.439 | <.001 | 0.558 | 0.368 |
| Physical Neglect | 0.204 | .010 | 0.041 | 0.262 |
| Emotional Abuse | 0.287 | .005 | 0.044 | 0.265 |
| Vulnerable Narcissism | 0.368 | <.001 | 0.403 | 1.036 |
| Physical Neglect | 0.154 | .078 | 0.228 | 0.105 |
| Emotional Abuse | 0.210 | .019 | 0.018 | 0.201 |
| Vulnerable Narcissism | 0.368 | <.001 | 0.403 | 1.036 |
| Absorption ~ |  |  |  |  |
| Vulnerable Narcissism | 0.373 | <.001 | 0.371 | 0.959 |
| Physical Neglect | 0.166 | .058 | -0.005 | 0.207 |
| Emotional Neglect | -0.191 | .018 | -0.147 | -0.015 |
| Sexual Abuse | 0.151 | .075 | -0.010 | 0.182 |
| Desire for thinness ~ |  |  |  |  |
| Vulnerable Narcissism | 0.310 | .001 | 0.748 | 3.138 |
| Bulimia ~ |  |  |  |  |
| Vulnerable Narcissism | 0.318 | <.001 | 0.724 | 2.457 |
| Depersonalization | 0.279 | .087 | -0.098 | 1.500 |
| Body Dissatisfaction ~ |  |  |  |  |
| Absorption | 0.353 | .003 | 0.596 | 2.790 |
| Vulnerable Narcissism | 0.364 | <.001 | 1.592 | 4.371 |
| Grandiose Narcissism | -0.160 | .032 | -3.053 | -0.132 |
